# Navigating Non‐Response: Prognosis and Strategies in Chronic Spontaneous Urticaria Management

**DOI:** 10.1111/ijd.17653

**Published:** 2025-01-09

**Authors:** Emek Kocatürk, Torsten Zuberbier

**Affiliations:** ^1^ Institute of Allergology Charité—Universitätsmedizin Berlin, Corporate Member of Freie Universität Berlin and Humboldt‐Universität zu Berlin Berlin Germany; ^2^ Department of Immunology and Allergology Fraunhofer Institute for Translational Medicine and Pharmacology (ITMP) Berlin Germany; ^3^ Department of Dermatology Bahcesehir University School of Medicine Istanbul Turkey

**Keywords:** angioedema, inflammatory diseases, treatment, urticaria

The study by Watanabe et al. [1], published in the February issue of the *International Journal of Dermatology*, on the prognosis of chronic spontaneous urticaria (CSU) patients who exhibit an inadequate response to omalizumab (OMA) is a significant contribution to understanding this challenging patient subgroup. The authors' findings offer valuable insights into prognostic determinants and therapeutic strategies, potentially aiding clinicians in optimizing the management of refractory CSU.

A pivotal observation from this study is that 58.3% of patients with an initial inadequate response to OMA achieved a favorable prognosis at 12 months. At first glance, this good prognosis could be attributed to the usage of higher doses of omalizumab since the international urticaria guidelines suggest increasing the dose or narrowing the intervals of omalizumab treatment when there is inadequate response to treatment with 300 mg/month [2]. However, the authors did not find an association between continuing omalizumab treatment and good prognosis in the multivariate analysis. This is most probably due to the fact that updosing omalizumab was not possible in Japan. Nevertheless, in clinical trials, more than half of the patients who had not responded to omalizumab 300 mg/month by Week 12 responded between Weeks 13 and 24 [3]. Therefore, a proportion of the patients who continued the same dose of omalizumab might have responded to treatment during follow‐up. The authors reported a positive association with good prognosis and the implementation of immunosuppressants and a negative association with systemic corticosteroids. This aligns with existing guidelines that advocate for minimizing corticosteroid use due to its long‐term adverse effects and exploring other options like cyclosporine‐A (cs‐A) for refractory cases.

The identified good prognostic factors were shorter CSU duration, concomitant angioedema (AE), low serum IgE (≤ 100 IU/mL) prior to OMA, elevated eosinophil counts (≥ 100/mm^3^) post‐OMA, and higher urticaria control test (UCT) scores pre‐ and post‐OMA (Figure 1).

**FIGURE 1:**
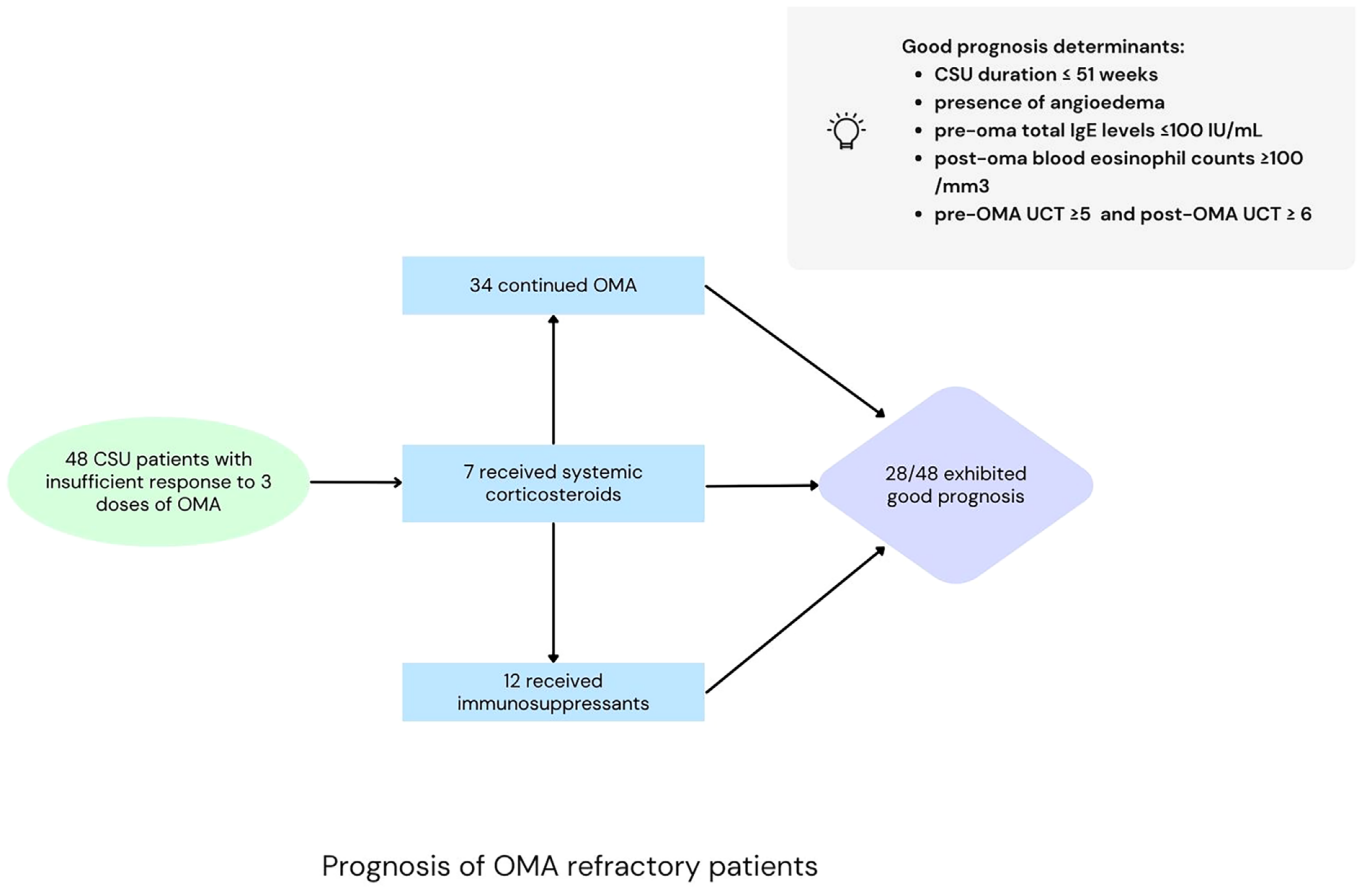
Prognosis of OMA refractory patients and good prognosis determinants.

Interestingly, the study highlights that AE, less frequently observed in Japanese patients than in Western patients, emerged as a good prognostic factor. This finding contrasts with previous reports where AE was often associated with poor outcomes [4]. The lower frequency of AE in Japanese CSU patients might reflect genetic or environmental differences, and its association with favorable prognosis in this cohort provides a novel perspective.

Moreover, the study underscores the importance of patient‐reported outcome measures like the urticaria control test (UCT) in evaluating disease control and tailoring management strategies [5]. UCT scores, which reflect the patient's perspective on disease control, emerged as a critical predictor of prognosis in this study. These tools should be routinely integrated into clinical practice to optimize individualized care and track treatment effectiveness.

While the study provides valuable insights, its retrospective design and modest sample size limit the generalizability of its findings. Additionally, variations in treatment protocols and healthcare systems across regions may affect outcomes, particularly the unavailability of up‐dosed or compressed‐interval OMA regimens in Japan.

Future studies should aim to validate these findings in larger, prospective cohorts, and explore the molecular and immunological mechanisms underpinning the identified prognostic factors. The potential role of combination therapies, such as OMA with CsA, warrants further investigation to optimize outcomes for refractory patients.

This study underscores the substantial burden of CSU, particularly in patients with suboptimal treatment responses. The findings highlight the importance of early diagnosis and intervention, given the association of shorter disease duration with better prognosis. Furthermore, the lack of a robust response to OMA in a subset of patients emphasizes the need for alternative therapies such as remibrutinib, dupilumab, and barzolvolimab and convenient biomarkers to guide treatment selection.

## Conflicts of Interest

The authors declare no conflicts of interest.
